# An Editors Perspective on Digital Pathology

**DOI:** 10.1155/2014/565437

**Published:** 2014-12-29

**Authors:** Stanley Cohen

**Affiliations:** Center for Biophysical Pathology, Rutgers NJMS, USA

## Abstract

There is currently a major disconnection in the publishing universe not only for retention, transmission, and analysis of digital images in pathology but also for new and emerging computationally intensive imaging methodologies. Many authors gravitate towards engineering journals, which are not usually read by pathologists or, for that matter, most biomedical investigators. The pathobiological application shown is often a proof-of-concept rather than a sophisticated investigative study. Papers that appear in pathology journals have the opposite problem; they are sketchy about the physical/engineering side of the work and do not attract many readers from the engineering and physics community. Nature methods partially bridge this gap, but it is more about the technology than the application of this technology for novel scientific discovery. For this reason, there is a need for a journal that can attract both kinds of authors (and readers) and serve as an integrative focus for work in these fields. Our evolving work towards this goal will be discussed.

